# *Clitoria ternatea* Flower Extract Attenuates Postprandial Lipemia and Increases Plasma Antioxidant Status Responses to a High-Fat Meal Challenge in Overweight and Obese Participants

**DOI:** 10.3390/biology10100975

**Published:** 2021-09-28

**Authors:** Thavaree Thilavech, Sirichai Adisakwattana, Pilailak Channuwong, Korntima Radarit, Kanthida Jantarapat, Kittisak Ngewlai, Nantarat Sonprasan, Charoonsri Chusak

**Affiliations:** 1 Department of Food Chemistry, Faculty of Pharmacy, Mahidol University, Bangkok 10400, Thailand; Thavaree.thi@mahidol.edu; 2 Phytochemical and Functional Food Research Unit for Clinical Nutrition, Department of Nutrition and Dietetics, Faculty of Allied Health Science, Chulalongkorn University, Bangkok 10330, Thailand; Sirichai.a@chula.ac.th (S.A.); 6177051537@student.chula.ac.th (P.C.); 3 Program in Doctor of Pharmacy, Faculty of Pharmacy, Mahidol University, Bangkok 10400, Thailand; korntima.rad@student.mahidol.ac.th (K.R.); Kanthida.jan@student.mahidol.ac.th (K.J.); kittisak.nge@student.mahidol.ac.th (K.N.); nantarat.son@student.mahidol.ac.th (N.S.)

**Keywords:** anthocyanins, *Clitoria ternatea*, high-fat meal, postprandial lipemia, antioxidant status

## Abstract

**Simple Summary:**

Supplementation of high-fat meals with edible plants is the principal strategy to control postprandial dysmetabolism and inflammation. This study demonstrated that consumption of *Clitoria ternatea* flower extract (CTE) decreased postprandial serum triglyceride and serum free fatty acids, and improved plasma antioxidant status and glutathione peroxidase activity responses to a high-fat meal challenge in overweight and obese participants. However, CTE could not reduce the effect of HF meal-induced increase in postprandial glycemia and the level of pro-inflammatory cytokines. The findings of the present study suggest that CTE may be used as an effective ingredient to suppress postprandial lipemia and improve the antioxidant status in overweight and obese individuals that frequently consume HF diets.

**Abstract:**

High-fat (HF) meal-induced postprandial lipemia, oxidative stress and low-grade inflammation is exacerbated in overweight and obese individuals. This postprandial dysmetabolism contributes to an increased risk of cardiovascular disease and metabolic disorders. *Clitoria ternatea* flower extract (CTE) possesses antioxidant potential and carbohydrate and fat digestive enzyme inhibitory activity in vitro. However, no evidence supporting a favorable role of CTE in the modulation of postprandial lipemia, antioxidant status and inflammation in humans presently exists. In the present study, we determine the effect of CTE on changes in postprandial glycemic and lipemic response, antioxidant status and pro-inflammatory markers in overweight and obese men after consumption of an HF meal. Following a randomized design, sixteen participants (age, 23.5 ± 0.6 years, and BMI, 25.7 ± 0.7 kg/m^2^) were assigned to three groups that consumed the HF meal, or HF meal supplemented by 1 g and 2 g of CTE. Blood samples were collected at fasting state and then at 30, 60, 90, 120, 180, 240, 300 and 360 min after the meal consumption. No significant differences were observed in the incremental area under the curve (iAUC) for postprandial glucose among the three groups. Furthermore, 2 g of CTE decreased the iAUC for serum triglyceride and attenuated postprandial serum free fatty acids at 360 min after consuming the HF meal. In addition, 2 g of CTE significantly improved the iAUC for plasma antioxidant status, as characterized by increased postprandial plasma FRAP and thiol levels. Postprandial plasma glutathione peroxidase activity was significantly higher at 180 min after the consumption of HF meal with 2 g of CTE. No significant differences in the level of pro-inflammatory cytokines (interleukin-6, interleukin-1*β* and tumor necrosis factor-α) were observed at 360 min among the three groups. These findings suggest that CTE can be used as a natural ingredient for reducing postprandial lipemia and improving the antioxidant status in overweight and obese men after consuming HF meals.

## 1. Introduction

A dramatic rise in the prevalence of overweight status and obesity has been reported among all age groups in developed as well as low/middle-income countries [1]. Obesity, a state of pathological increase in the amount of adipose tissue and accumulation of excessive body fat mass, is recognized as a risk factor for developing non-communicable diseases (NCDs), such as type 2 diabetes, hypertension, hyperlipidemia and cardiovascular diseases (CVDs) [2]. Interestingly, not all people meeting the criteria for obesity demonstrate poor metabolic complications; they are considered to present with a metabolically healthy obesity that is in transition into developing into metabolically unhealthy obesity [3].

In obesity, excessive fat accumulation in adipose tissues promotes chronic low-grade inflammation related to produce a variety of pro-inflammatory cytokines, such as interleukin-6 (IL-6), interleukin-1*β* (IL-1*β*) and tumor necrosis factor-α (TNF-α) [4]. Many studies suggest that chronic inflammation could have a serious role in insulin resistance, which precedes the onset of type 2 diabetes in adults [5]. Chronic inflammatory processes could induce the generation of free radicals which may further lead to insulin resistance by impairing insulin signaling and sensitivity [6,7]. Interestingly, overexpression of oxidative process damages biological molecules and decreases the activity of endogenous antioxidant enzymes, such as glutathione peroxidase (Gpx), catalase and glutathione reductase [8].

Abnormalities in postprandial triglycerides are considered an independent CVD risk factor [9]. It is well-known that macronutrients such as carbohydrates and fats are the main modulators of abnormal postprandial lipemic responses [10]. In particular, the consumption of high-fat (HF) meals markedly increases postprandial triglycerides and further promotes the generation of free radicals [11,12]. Consequently, lipid peroxidation generated from polyunsaturated fatty acids proceeds by free radical chain reaction, resulting in the formation of malondialdehyde (MDA), the most mutagenic by-product [13]. To this end, decreasing the magnitude of postprandial lipemia, as well as increasing antioxidant capacity, has been the target of food-based nutritional interventions.

Recent findings indicate that the suppression of postprandial lipemic responses and the improvement of oxidative stress could be achieved by consumption of edible plants [14,15,16]. For example, the consumption of grape seed extract and strawberry with a high-fat diet could attenuate the postprandial rise in blood triglyceride levels and increase the postprandial antioxidant status in humans [14,15]. In addition, consumption of tart cherry, an anthocyanin-rich foods, by healthy individuals on a high-fat diet significantly reduces postprandial triglycerides and increases plasma antioxidant capacity [16]. Furthermore, ingestion of strawberry beverage together with a high-carbohydrate, moderate-fat meal causes a reduction in postprandial inflammatory response such as high-sensitivity C-reactive protein and interleukin-6 (IL-6) with a concomitant increase in antioxidant capacity in overweight and obese participants [17]. Therefore, supplementing high-fat meals with edible plants may be the principal strategy for improving postprandial hyperlipidemia, oxidative stress and inflammation response.

*Clitoria ternatea* L. (butterfly pea) flower is an edible plant belonging to the Fabaceae family that is widely grown in tropical and temperate regions worldwide. The blue color of *Clitoria ternatea* (*C. ternatea*) flower is used as a natural colorant for the preparation of various foods and beverages. This herbaceous plant has been used in traditional Ayurvedic medicine for centuries as a memory enhancer, anti-stress, anti-depressant, anti-convulsant, anti-bacterial, anti-inflammation and sedative agent [18]. The flower of *C. ternatea* contains a variety of phytochemical compounds, such as phenolic acids and flavonoids. The major colorants of this flower are anthocyanins, derived from delphinidin, namely, ternatin anthocyanins such as A1–A3, B1–B4, C1 and D1–D3 [19]. Our group previously reported that the *C. ternatea* flower extract (CTE) inhibits fructose- and methylglyoxal-induced protein glycation and oxidative damage to bovine serum albumin in vitro [20,21]. Recent studies have also demonstrated the biological effect of *C. ternatea* related to carbohydrate and lipid metabolism [22,23,24]. For example, CTE inhibits adipogenesis and reduces the accumulation of triglyceride in 3T3-L1 preadipocytes by downregulating adipogenic gene expression [22]. Furthermore, CTE inhibits α-amylase and intestinal α-glucosidase activity in vitro [23,24]. In food application, replacing wheat flour with CTE was shown to decrease the formation of thiobarbituric acid reactive substances (TBARs) in sponge cake [25]. CTE could also reduce starch hydrolysis, thereby decreasing the release of glucose from various types of flour, including potato, cassava, rice, corn, wheat and glutinous rice, in in vitro-simulated gastrointestinal digestion [26]. In the context of glycemic response, acute consumption of CTE beverage containing disaccharides (sucrose) was shown to decrease postprandial glucose concentration and improve the antioxidant status in healthy participants [27].

Nevertheless, the effect of co-ingestion of CTE with a high-fat meal on postprandial lipemic and pro-inflammatory marker responses in humans has never been investigated. Therefore, we aimed to determine the effect of CTE on postprandial glycemia, triglyceride, free fatty acids (FFA), antioxidant status and inflammatory markers following HF meal intake by overweight and obese men.

## 2. Materials and Methods

### 2.1. Chemicals

Folin–Ciocalteu reagent, 2,4,6-tripyridy-S-Triazine (TPTZ), malondialdehyde (MDA), butylated hydroxytoluene (BHT) and thiobabituric acid (TBA) were obtained from Sigma-Aldrich (St. Louis, MO, USA), whereas 5, 5′-dithiobis-(2-nitrobenzoic acid) (DTNB) and trichloroacetic acid were purchased from Merck (Darmstadt, Germany).

### 2.2. Preparation of C. ternatea Extract (CTE)

Dried *C. ternatea* flowers were purchased in 2018 from a local herbal market, Bangkok, Thailand. An exsiccate of *C. ternatea* flowers was authenticated at the Princess Sirindhorn Plant Herbarium, Plant Varieties Protection Division, Department of Agriculture, Bangkok, Thailand, and was deposited under Voucher specimen ID: BKU066793. The extraction process was conducted according to a previous report by Chusak et al. [27]. Briefly, the powder of dried flowers was extracted by distilled water at a 1:20 (*w*/*v*) ratio. After filtering the solution with Whatman No. 1, the frozen solution was placed in a freeze-dryer GFD-30S and dried at −30 °C, at a pressure of 0.15 mbar for 35 h (GRT, Grisrianthong. Co., Ltd., Ratchaburi, Thailand). The powder of CTE was kept in a laminated aluminum foil vacuum bag and stored at −20 °C until analysis. The total phenolic content (TPC) and total anthocyanins were determined by the Folin–Ciocalteu method and pH-differential method, respectively [21]. TPC and total anthocyanins were 50.19 ± 0.86 mg gallic acid equivalent/g extract and 0.87 ± 0.13 mg delphinidin-3-glucoside equivalent/g extract, respectively.

### 2.3. Participants

This study was conducted according to the Declaration of Helsinki guidelines and was approved by the office of Ethics Review Committee for Research Involving Human Research Subjects, Human Science Group, Chulalongkorn University, and the office of Faculty of Dentistry/Faculty of Pharmacy, Mahidol University Institutional Review Board (COA No. 203/2562 and COA.NO.MU-DT/PY-IRB 2019/061.0309, respectively). This trial is registered at the Thai Clinical Trials Registry (TCTR) under the identifier TCTR20200114006. All participants provided written informed consent and their identities were kept confidential. For inclusion criteria, only overweight or obese men aged 20−40 years with a body mass index (BMI) between 23 and 30 kg/m^2^ (according to the WHO Asian BMI classification [28]), fasting blood glucose <100 mg/dL, blood urea nitrogen (BUN) between 8 and 24 mg/dL, creatinine between 0.84 and 1.21 mg/dL, aspartate transaminase (AST) between 8 and 48 U/L, alanine transaminase (ALT) between 7 and 55 U/L and blood pressure <120/80 mmHg were eligible for participation. The exclusion criteria included having any chronic diseases, such as diabetes mellitus, hypercholesterolemia, liver or kidney diseases, food allergies (cow’s milk, butter, wheat and gluten), taking any medications or supplements and smoking or alcohol consumption. All eligible individuals also underwent a screening procedure, as a part of which blood biochemistry was collected and analyzed by the Health Sciences Service Unit, Faculty of Allied Health Sciences, Chulalongkorn University. They also took part in a screening interview as a part of which medical and dietary history was obtained by the researchers.

The sample size was calculated based on a previous study focusing on the change of plasma antioxidant capacity presented by oxygen radical absorbance capacity (ORAC) after the consumption of a high-fat meal with a freeze-dried blueberry powder [29]. A minimum sample size of 15 participants per group was determined as sufficient for achieving a confidence level of 95% (α = 5%) and a power of 80%. Anticipating 20% attrition, the sample size was increased to 18 participants. Thus, 19 individuals were recruited for screening and only 16 completed the study.

### 2.4. Study Design

This clinical trial was conducted at the Department of Nutrition and Dietetics, Faculty of Allied Health Sciences, Chulalongkorn University, Bangkok, Thailand. A randomized, single-blind, crossover design with one week wash-out period was adopted. Prior to commencing the study, participants underwent BMI, fasting plasma glucose, triglyceride, total cholesterol, liver and kidney function tests. All participants were instructed to maintain habitual dietary and physical activity patterns during the study period.

**Figure 1:**
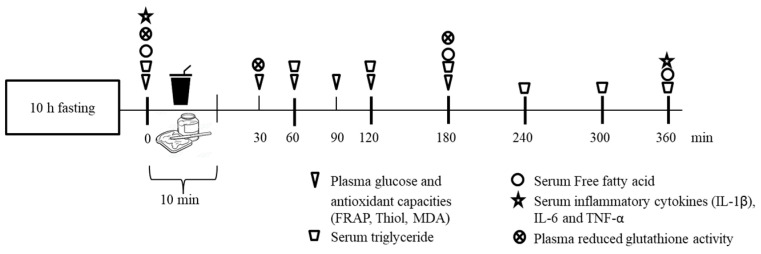
Intervention scheme.

Before arrival on each testing day, participants were instructed to abstain from alcohol, high-fat food, antioxidant-rich food (such as berries and citrus fruits) and dietary supplements consumption, as well as vigorous exercise, for at least 24 h. After overnight fasting for 10 h, participants visited the study center and, after resting for 10 min, they had a venous catheter inserted into the left arm by a registered nurse. A randomized table with intervention for each individual was generated before the start of the experiment using an online random number generator (https://www.random.org [accessed on 10 October 2019]). The experimental design involved three intervention groups, whereby assigned participants consumed a high-fat (HF) meal, an HF meal plus 1 g of CTE, or an HF meal plus 2 g of CTE, respectively. Participants were asked to consume the meal within 10 min. Blood samples were collected at fasting state and at 30, 60, 90, 120, 180, 240, 300 and 360 min after the meal consumption by a clot activator tube and were subjected to triglyceride, free fatty acid and inflammatory cytokine analysis. The plasma for antioxidant capacity and blood glucose analysis was collected by a blood collecting tube with EDTA and sodium fluoride, respectively. Blood samples were immediately centrifuged at 3000 rpm for 10 min at 4 °C. The plasma and serum samples were separated and kept at −20 °C until required for further analysis (Figure 1). During this 6 h period, participants were permitted to drink up to 1 L of water.

### 2.5. Intervention

According to the Thai Dietary Recommendation of Intakes (Thai DRI) established by the Department of Health, Ministry of Public Health of Thailand in 2020, the energy requirement for males aged 19–30 years is 2260 kcal/day. The HF meal provided approximately 720 kcal with 50:41:9 caloric distribution of fat, carbohydrate and protein. The meal consisted of three slices of white bread with 5 g of condensed milk and 30 g of butter and 240 mL of whole milk containing 10 g of a commercial medical food Ensure^®^ (Abbott Laboratories Limited, Abbott Park, IL, USA) as a beverage with or without CTE (1 g or 2 g). The meals were freshly prepared by the researchers in the morning of each test day.

### 2.6. Plasma Glucose, Serum Triglyceride and Free Fatty Acid (FFA)

Plasma glucose and serum triglyceride concentration were measured using Glucose and Triglycerides liquicolor reagent (Human^®^ GmbH, Wiesbaden, Germany), respectively. Serum free fatty acids (FFA) levels were determined using non-esterified fatty acid enzymatic cycling assay kit (BIOBASE, Jinan, China).

### 2.7. Plasma Ferric Reducing Antioxidant Power (FRAP)

The FRAP value representing antioxidant power was determined according to the method described previously [30]. Plasma was diluted to 1:2 with 0.1 M phosphate buffer saline (PBS, pH of 7.4). The FRAP reagent contained 0.3 M sodium acetate buffer (pH of 3.6), 10 mM TPTZ in 40 mM HCl and 20 mM FeCl_3_ at a 10:1:1 (*v*/*v*) ratio, respectively. The working reagent was freshly prepared and warmed at 37 °C before use. Next, 10 µL of diluted plasma was mixed with 90 µL of FRAP reagent and was incubated at room temperature for 5 min in darkness. The absorbance was determined at 595 nm. FeSO_4_ was used as standard to generate the calibration curve for FRAP value calculation and the results were expressed as mM FeSO_4_.

### 2.8. Plasma Thiol

The plasma thiol concentration was determined by Ellman’s assay with minor modifications [30]. The 1:10 diluted plasma (90 µL) was mixed with 130 µL of 2.5 mM DTNB in PBS and was incubated at room temperature for 15 min. The absorbance was measured at 412 nm. The plasma thiol concentration was calculated using a standard L-cysteine curve and was expressed as mM L-cysteine.

### 2.9. Plasma Lipid Peroxidation

Lipid peroxidation was determined by thiobarbituric acid-reactive-substances assay (TBARS) [31], which measures MDA as a secondary product of lipid peroxidation. Briefly, 200 µL of plasma was mixed with an equal amount of 15% (*w*/*v*) trichloroacetic acid and 30 µL of 0.25 mM BHT in ethanol. The mixture was centrifuged at 13,000 rpm for 10 min to precipitate protein and the supernatant (200 µL) was collected and mixed with 0.375% (*w*/*v*) TBA (200 µL). The reaction was heated at 95 °C for 10 min. After cooling down, the absorbance was measured at 532 nm and the plasma MDA concentration was calculated using a standard curve of MDA.

### 2.10. Plasma Glutathione Peroxidase Activity

The glutathione peroxidase activity was determined using a glutathione peroxidase assay kit (Cayman Chemical, Ann Arbor, MI, USA) according to the manufacturer’s protocol. The plasma samples were obtained with a blood collecting tube with EDTA as anticoagulant and were diluted by 1:2 with PBS before analysis.

### 2.11. Plasma Inflammatory Cytokines

The circulating pro-inflammatory cytokine (IL-1*β*, IL-6 and TNF-α) levels were determined using an enzyme-linked immunosorbent assay (ELISA) kit according to the manufacturer’s manual (BIOBASE, Shandong, China).

### 2.12. Statistical Analysis

All values are expressed as mean ± SEM. Postprandial incremental areas under the curve (iAUC) for glucose, triglyceride, FRAP, thiol and MDA were analyzed using the Trapezoidal method. The Kolmogorov–Smirnov test was performed to determine whether the data were normally distributed. Repeated measures one-way analysis of variance (ANOVA) followed by Duncan’s multiple range post hoc test was conducted to compare the effect of interventions (treatment, time and treatment × time interaction). All statistical analysis were performed using SPSS version 22.0 (Chicago, IL, USA) and *p*-value < 0.05 was considered statistically significant.

## 3. Results

### 3.1. Participants

**Figure 2:**
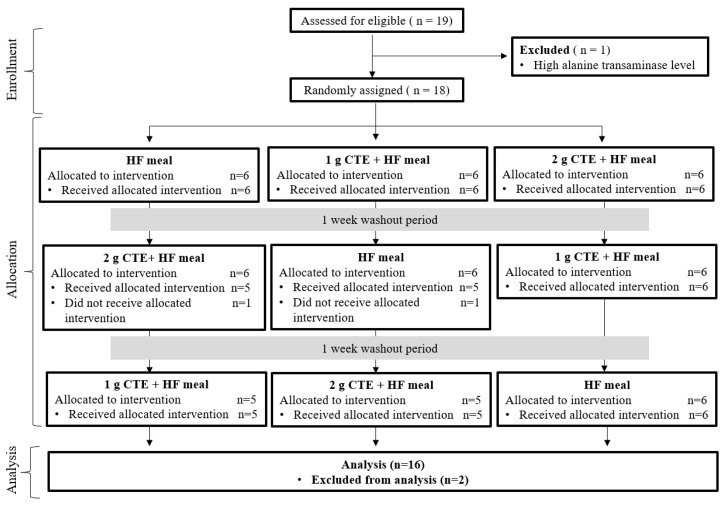
Consolidating Standards of Reporting (CONSORT) flow diagram of selection of participants.

At the start of the study, 19 individuals were recruited for screening, but one participant was excluded due to high aspartate transaminase levels. The remaining 18 participants were randomly assigned to the three intervention groups, but only 16 (9 overweight and 7 obese participants) completed the study. Two participants who did not receive intervention were excluded from analysis. The enrollment and allocation information are shown in Figure 2, while participant characteristics are reported in Table 1. The mean age of the participants who completed the study was 23.5 ± 0.6 years and their average BMI was 25.7 ± 0.7 kg/m^2^. The pre-screening blood biochemical parameters were checked to ensure that the participants were eligible to take part in the study.

### 3.2. Postprandial Plasma Glucose Concentration

**Figure 3:**
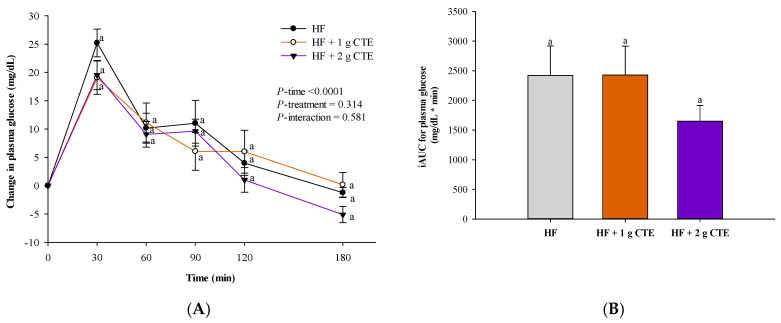
Changes in (**A**) postprandial plasma glucose concentration and (**B**) incremental area under the curve (iAUC) in overweight and obese adults after consuming the HF meal with 1 g and 2 g of *Clitoria ternatea* extract (CTE). The fasting plasma glucose concentration was 88.3 ± 2.8 mg/dL for the HF meal, 86.2 ± 2.5 mg/dL for the HF meal + 1 g CTE and 84.8 ± 2.8 mg/dL for the HF meal + 2 g CTE. Values are means ± SEM, *n* = 16. Different letters are significantly different.

The postprandial changes in plasma glucose concentration after consuming HF supplemented with CTE are shown in Figure 3A. There were significant incremental postprandial plasma glucose changes in all three groups (*p* < 0.0001 for time effect) with no statistically significant interaction of treatment and time × treatment effect (*p* > 0.05). The HF meal challenge induced an increase in postprandial glucose concentration that peaked at 30 min. There were no significant differences in the postprandial glucose changes between individuals that consumed the HF meal plus CTE (1 g and 2 g) at any time point when compared to those given HF meal only. In addition to the postprandial results, no significant effects were observed in the iAUC of plasma glucose among the three groups (Figure 3B).

### 3.3. Postprandial Serum Triglyceride and FFA Concentration

**Figure 4:**
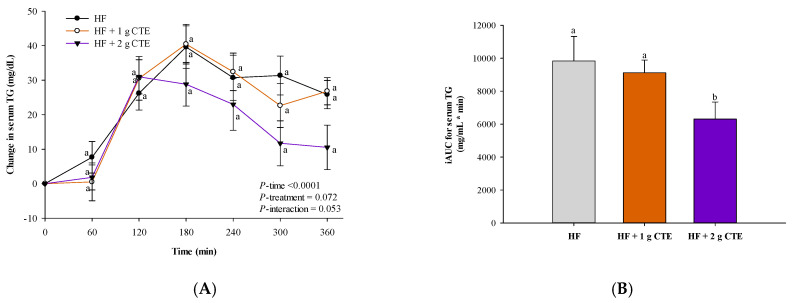
Changes in (**A**) postprandial serum triglyceride (TG) concentration and (**B**) incremental area under the curve (iAUC) in overweight and obese adults after consuming the HF meal with 1 g and 2 g of *Clitoria ternatea* extract (CTE). The fasting serum triglyceride concentration was 106.2 ± 12.8 mg/dL for the HF meal, 108.8 ± 9.5 mg/dL for the HF meal + 1 g CTE and 101.5 ± 13.1 mg/dL for the HF meal + 2 g CTE. Values are means ± SEM, *n* = 16. Different letters are significantly different (*p* < 0.05).

In all three groups, significant changes in incremental postprandial serum triglyceride were observed (*p* < 0.0001 for time effect) with no statistically significant interaction of treatment and time × treatment effect (Figure 4A). Postprandial triglyceride concentration tended to be lower at 300 and 360 min after consuming the HF meal supplemented with CTE (2 g). However, the decrease was statistically insignificant at those time points. The HF meal cooperated with 2 g of CTE resulted in a significant reduction in the serum triglyceride iAUC when compared to the HF meal (Figure 4B).

**Figure 5:**
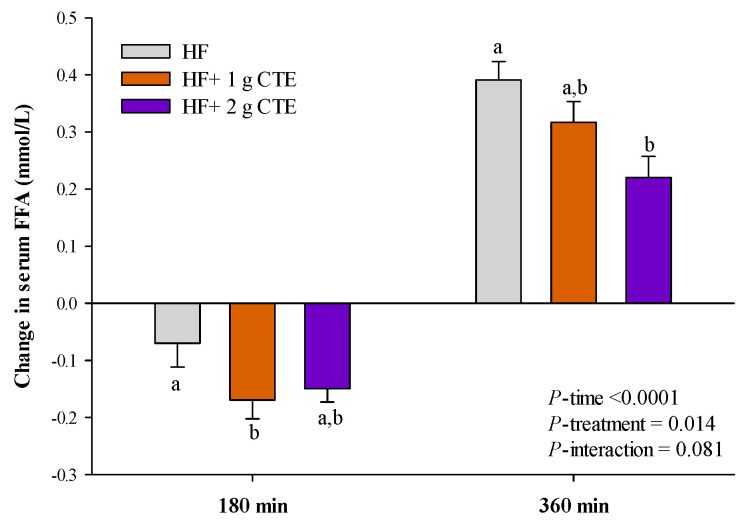
Changes in serum free fatty acid (FFA) concentration in overweight and obese adults after consuming the HF meal with 1 g and 2 g of *Clitoria ternatea* extract (CTE). The fasting serum free fatty acid (FFA) was 0.569 ± 0.079 mmol/L for the HF meal, 0.619 ± 0.066 mmol/L for the HF meal + 1 g CTE and 0.586 ± 0.058 mmol/L for the HF meal + 2 g CTE, respectively. Values are means ± SEM, *n* = 16. Different letters are significantly different (*p* < 0.05).

Compared to the values at fasting state, the serum FFA concentration decreased at 180 min and then increased at 360 min following the HF meal. Interestingly, 2 g of CTE significantly decreased postprandial serum FFA concentration at 360 min compared to HF and HF + 1 g CTE meals (Figure 5).

### 3.4. Postprandial Antioxidant Status

**Figure 6:**
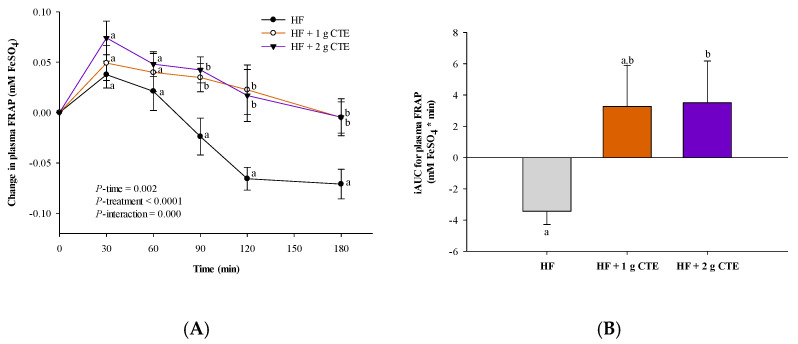

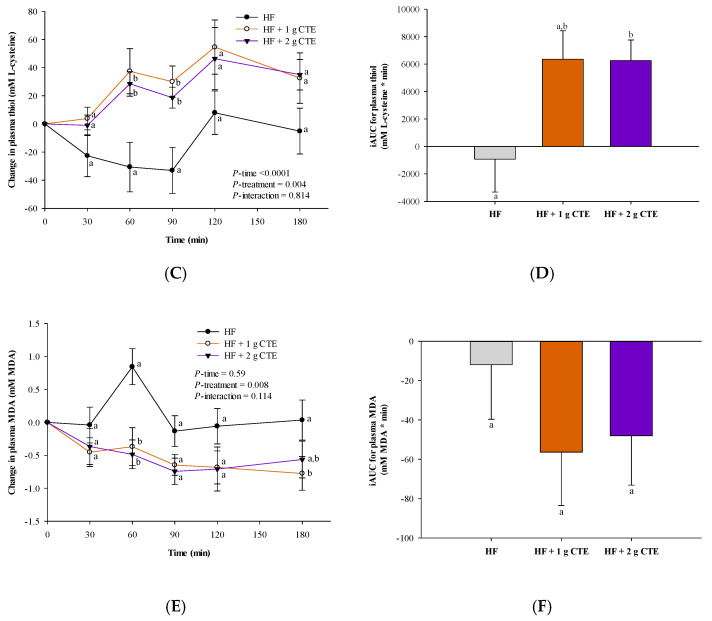
Changes in postprandial (**A**) plasma ferric reducing antioxidant power (FRAP), (**C**) thiol and (**E**) malondialdehyde (MDA) concentration and the incremental area under the curve (iAUC) of (**B**) FRAP, (**D**) thiol and (**F**) MDA concentration in overweight and obese adults who consumed the HF meal with 1 g and 2 g of *Clitoria ternatea* extract (CTE). The fasting plasma FRAP values were 0.60 ± 0.04 mM FeSO_4_ for the HF meal, 0.67 ± 0.04 mM FeSO_4_ for the HF meal + 1 g CTE and 0.63 ± 0.06 mM FeSO4 for the HF meal + 2 g CTE. The fasting plasma protein thiol concentration was 496.3 ± 33.0 mM L-cysteine for the HF meal, 479.4 ± 28.5 mM L-cysteine for the HF meal + 1 g CTE and 467.0 ± 21.5 mM L-cysteine for the HF meal + 2 g CTE. The fasting plasma MDA concentration was 9.54 ± 0.83 µM for the HF meal, 10.01 ± 0.34 µM for the HF meal + 1 g CTE and 9.70 ± 0.41 µM for the HF meal + 2 g CTE. Values are means ± SEM, *n* = 16. Different letters are significantly different (*p* < 0.05).

When compared to the values at fasting state, ingestion of the HF meal resulted in a decrease in the postprandial plasma FRAP level at 90, 120 and 180 min (Figure 6A). At the same time points, addition of CTE caused a significantly higher postprandial FRAP level when compared to the HF meal. The iAUC of FRAP also revealed significantly higher values for HF + 2 g CTE vs. HF meal alone (*p* < 0.05; Figure 6B).

The effects of the HF meal with CTE on postprandial plasma thiol concentration are illustrated in Figure 6C. Compared to the values at fasting state, HF meal consumption decreased postprandial plasma thiol concentration at 30, 60 and 90 min. Moreover, postprandial plasma thiol concentration after consumption of HF meal accompanied with 1 g and 2 g of CTE was significantly higher at 60 and 90 min than that of the HF meal alone (*p* < 0.05). As shown in Figure 6D, the iAUC for postprandial plasma thiol in the of HF + 2 g CTE group was higher than that of the HF group (*p* < 0.05).

The effects of CTE on postprandial plasma MDA concentration after consumption of the HF meal are presented in Figure 6E. As can be seen from the graph, HF meal intake induced a slight increase in postprandial plasma MDA at 60 min. However, this postprandial effect was attenuated by adding 1 g and 2 g of CTE to the HF meal (*p* < 0.05). On the other hand, there were no significant differences in the iAUC for postprandial plasma MDA concentration among all three groups (Figure 6F).

### 3.5. Postprandial Plasma Glutathione Peroxidase (Gpx) Activity

**Figure 7:**
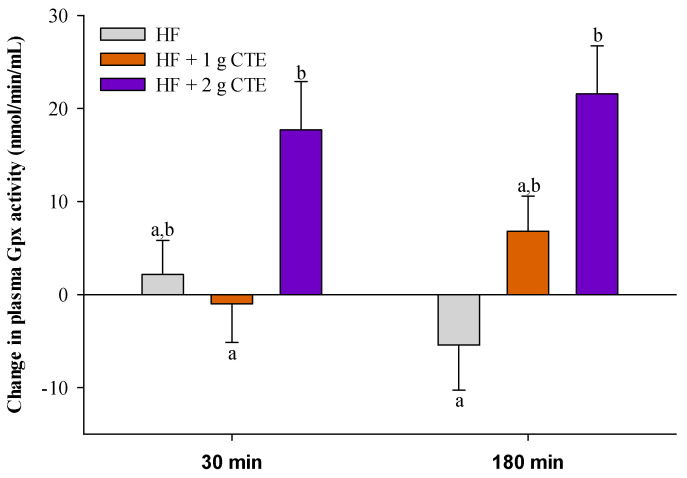
Changes in the activity of plasma glutathione peroxidase (Gpx) in overweight and obese adults who consumed the HF meal with 1 g and 2 g of *Clitoria ternatea* extract (CTE) at 30 and 180 min. The fasting plasma Gpx activity was 142.0 ± 8.8 nmol/min/mL for the HF meal, 142.0 ± 9.7 nmol/min/mL for the HF meal + 1 g CTE and 154.8 ± 5.5 nmol/min/mL for the HF meal + 2 g CTE. Values are means ± SEM, *n* = 16. Different letters are significantly different (*p* < 0.05).

HF meal ingestion caused a reduction in plasma Gpx activity at 180 min when compared to the fasting state (Figure 7). Moreover, addition of CTE (2 g) led to an increase in postprandial plasma Gpx activity at 180 min relative to HF meal alone (*p* < 0.05).

### 3.6. Postprandial Serum Pro-Inflammatory Cytokines

**Figure 8:**
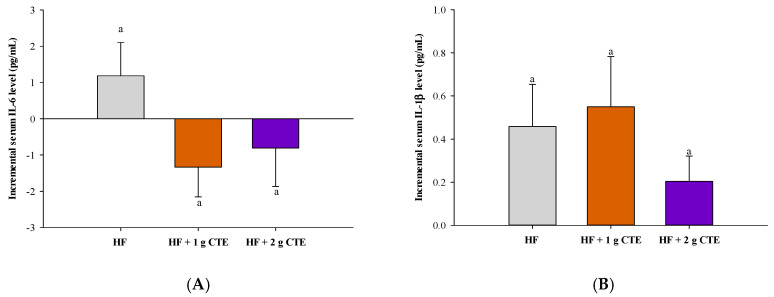

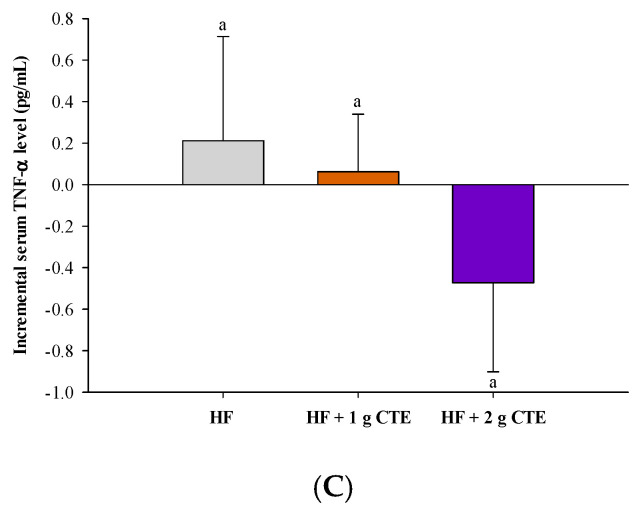
Changes in the level of postprandial serum (**A**) interleukin (IL)-6, (**B**) IL-1*β* and (**C**) tumor necrosis factor (TNF)-α in overweight and obese adults who consumed the HF meal with 1 g and 2 g of *Clitoria ternatea* extract (CTE) at 360 min. The fasting serum IL-6 concentration was 34.86 ± 1.04 pg/mL for the HF meal, 34.32 ± 0.87 pg/mL for the HF meal + 1 g CTE and 35.70 ± 0.79 pg/mL for the HF meal + 2 g CTE, respectively. The fasting serum IL-1*β* concentration was 29.51 ± 1.75 pg/mL for the HF meal, 25.82 ± 1.76 pg/mL for the HF meal + 1 g CTE and 29.05 ± 4.77 pg/mL for the HF meal + 2 g CTE, respectively. The fasting serum level of TNF-α was 63.12 ± 1.43 pg/mL for the HF meal, 63.17 ± 3.02 pg/mL for the HF meal + 1 g CTE and 76.45 ± 3.54 pg/mL for the HF meal + 2 g CTE. Values are means ± SEM, *n* = 16. Different letters are significantly different (*p* < 0.05).

The postprandial serum pro-inflammatory cytokine levels, including interleukin (IL)-6, IL-1*β* and tumor necrosis factor (TNF)-α, after consuming the test meals are presented in Figure 8A–C, respectively. When compared to the values at fasting state, postprandial IL-6 and IL-1*β* concentrations increased at 360 min after consuming the HF meal, whereas the concentration of TNF-α remained unchanged. Although a slight reduction in the level of serum IL-6 and TNF-α was noted for HF + 1 g CTE and HF + 2 g CTE, and serum IL-1*β* was decreased only in the HF + 2 g CTE group, none of the differences among groups were statistically significant.

## 4. Discussion

Globally, there have been considerable changes in behavior and lifestyle characterized by an increased intake of energy-dense and high-fat foods. Recent epidemiological studies clearly demonstrated the link between overconsumption of HF foods and the risk of overweight- and obesity-associated chronic metabolic diseases, such as CVD and diabetes [32]. Specifically, an intake of HF meal causes postprandial lipemia leading to increased inflammatory and oxidative stress markers [6,33]. These responses have gained interest due to the association of postprandial triglyceride levels and the risk of CVD that have been demonstrated by recent reports. An increase in postprandial triglyceride levels are possibly even greater independent predictors of CVD than fasting triglyceride [34]. Our results showed that postprandial plasma glucose and serum triglyceride concentration were increased in overweight and obese men after consuming HF meal and postprandial lipemia was observed at 3 h after fat loading. This finding is consistent with the results reported by Clemente-Postigo et al., indicating that fat overload induces an increase in postprandial hypertriglyceridemia and chylomicron fraction in morbidly obese patients [35].

Several clinical studies have been conducted on the effects of plants containing polyphenols on postprandial lipemia. Their findings indicate that postprandial serum triglyceride exhibit a decreasing trend at 4–6 h following ingestion of raspberries or freeze-dried strawberry powder containing polyphenols and anthocyanins alongside a high-fat meal in adults [36,37]. In accordance with other reports, in the present study, CTE (2 g) suppressed the postprandial plasma triglycerides and FFA magnitude and peak time response to the HF meal. We attribute the inhibitory effects of phenolic acids, polyphenols and anthocyanins against pancreatic lipase to the ability of CTE to reduce postprandial triglyceride levels. It has been shown that delphinidin-3,5-glucoside, delphinidin-3-glucoside, malvidin-3β-glucoside, kaempferol, *p*-coumaric acid and six major ternatins (A1, A2, B1, B2, D1 and D2) are the main phytochemical compounds in CTE [18,21]. These compounds have been reported to inhibit pancreatic lipase activity that further blocks the hydrolysis of triglycerides into glycerol and free fatty acids [38]. The reduction in fat absorption through pancreatic lipase inhibition is known to assist with controlling postprandial hypertriglyceridemia as an independent predictor of CVD [39,40]. Therefore, CTE may help to prevent obesity and CVD through the suppression of postprandial hypertriglyceridemia. However, the relationship between the ingestion of CTE and CVDs remains unclear and this aspect should be further investigated.

Available evidence demonstrates that postprandial hyperglycemia/insulinemia and hyperlipidemia positively correlates with oxidative stress and inflammation [12]. A high-fat diet induces postprandial lipemia, leading to oxidative stress and inflammatory response through various mechanisms [11]. The previous study found that acute consumption of high-fat diets contributes to a significant increase in postprandial triglycerides accompanied with reduced postprandial plasma antioxidant activity [41]. In this study, consumption of a high-fat diet resulted in a decline in postprandial plasma antioxidant activity, as indicated by the reduction in plasma FRAP and protein thiol levels. An increase in postprandial plasma MDA (as an oxidative stress marker of lipid peroxidation) was also observed at 30 min after high-fat meal intake. The alteration of postprandial antioxidant activity was previously attributed to lipid peroxidation by the hydrogen abstraction or addition of an oxygen radical, resulting in the decomposition of polyunsaturated fatty acids [42]. In addition, presence of disulfide bonds in sulfhydryl groups was shown to act as an antioxidant defense mechanism [43]. The depletion of protein thiol indicates an increased oxidative protein damage, causing a decrease in plasma antioxidant activity. In the present study, ingestion of CTE with the HF meal improved postprandial plasma antioxidant capacity by increasing plasma FRAP and maintaining the plasma thiol levels. These findings are consistent with those yielded by our previous study demonstrating the effect of CTE beverage on postprandial plasma antioxidant capacity in healthy subjects. Drinking CTE (1 g or 2 g) with and without a sucrose-containing beverage elevated plasma FRAP, oxygen radical absorbance capacity (ORAC), trolox equivalent antioxidant capacity (TEAC) and protein thiol, while decreasing the MDA levels [27]. According to Chusak et al., the antioxidant capacity of blood increases after consuming beverages containing CTE because of the antioxidant activity of phytochemical compounds presented in CTE [27]. Our previously reported findings support the suggestion that CTE demonstrates strong antioxidant activity toward free radical scavengers [20].

Glutathione peroxidase (Gpx), known as a selenium-dependent enzyme, is one of the most essential endogenous antioxidant enzymes in the human body, as it helps to eliminate hydrogen peroxides and other organic peroxides, such as lipid peroxides [44]. In addition to the antioxidant defense mechanism, Gpx decomposes peroxide molecules into water and competes with catalase for hydrogen peroxides, thereby reducing the degree of oxidative stress in cells and limiting tissue damage [44]. Several studies have shown that consumption of edible plants containing polyphenol compounds increases the activity of antioxidant enzymes in rats and humans [45,46,47]. In the present study, the consumption of the HF meal with CTE was shown to increase Gpx activity with a concomitant increase in plasma FRAP and thiol at 180 min. An increase in Gpx activity with decreasing protein oxidation by a phytochemical-rich plant was also observed in high-fat, high-fructose diet-induced obese rats [47]. Our findings suggest that the Gpx activity increase by CTE may be related to multiple mechanisms of action. We posit that CTE may exert a protective effect against high fat-induced oxidative damage to antioxidant defense enzymes through its antioxidant activity as a direct scavenger. Consequently, this action helps blood circulation to maintain high levels of antioxidant enzyme activity for preventing excessive generation of harmful reactive oxygen species. Moreover, CTE may directly activate the antioxidant response by promoting specific redox-sensitive transcription factors, such as activating protein 1 (AP-1) and NF-κB, leading to an increase in the mRNA expression of endogenous antioxidants, such as superoxide dismutase, catalase and Gpx [48,49].

Extant studies further indicate that HF meal intake is associated with higher pro-inflammatory cytokine levels through the activation of immune cells, leading to increased low-grade inflammation [50,51]. Interestingly, saturated fatty acids were found to induce the release of pro-inflammatory cytokines, such as IL-6, IL-1*β* and TNF-α, from immune cells into blood circulation, particularly in individuals with metabolic disorders [52]. In the study conducted by Herieka et al., the peak concentration of postprandial pro-inflammatory cytokines was observed 4–6 h after HF meal loading [53]. Similarly, in the current study, the alteration in the pro-inflammatory cytokine profile was noted at 6 h. It has been reported that the intake of phytochemical-rich plants has the capacity to suppress the rise in pro-inflammatory cytokine response to the HF meal. For instance, consumption of HF foods accompanied by a fruit juice resulted in a significant decrease in plasma cholesterol and triglyceride concentration in healthy overweight subjects with a concomitant reduction of inflammatory response mediated by IL-6 and TNF-α [54]. On the other hand, Davis et al. reported that an intake of polyphenol-rich cocoa could not improve postprandial inflammatory biomarkers (IL-6 and IL-1*β*) in subjects following HF meal intake [55]. According to Edirisinghe et al., serum concentration of IL-1*β* was not altered in individuals that consumed strawberry beverages alongside an HF meal [17]. However, consumption of CTE tended to counteract the HF meal effect on the level of pro-inflammatory cytokines. This finding may be a result of low doses of phytochemical compounds in CTE, which could not induce sufficient reduction in postprandial circulating pro-inflammatory cytokine levels following an HF meal.

The main contribution of the current study stems from its original design and its focus on postprandial effect of CTE on the lipemic response and antioxidant capacity after consumption of an HF meal. Specifically, we incorporated CTE into a beverage to mimic the traditional consumer behavior. However, as the sample size was small, the study findings cannot be generalized. Moreover, participants were aware of their group assignment because the colors of CTE and control beverages were different. Finally, only male participants were recruited for this study to avoid the effect of gender on the measured variations in blood biochemistry. To address these limitations, further studies are warranted, especially long-term trials involving both male and female individuals with cardiovascular risk factors.

## 5. Conclusions

This is the first study to demonstrate that acute consumption of an HF meal accompanied with CTE decreases postprandial serum triglycerides and FFA concentration in overweight and obese men. CTE also significantly improves plasma antioxidant status responses to the HF meal by increasing plasma FRAP, thiol and the activity of endogenous antioxidant enzyme, glutathione peroxidase. However, CTE could not reduce the effect of HF meal-induced increase in postprandial glycemia and the level of pro-inflammatory cytokines. These findings suggest that CTE may be used as an effective ingredient to suppress postprandial lipemia and improve the antioxidant status in overweight and obese individuals that frequently consume HF foods.

## Figures and Tables

**Table 1:** Characteristics of the participants.

Characteristics	Mean ± SEM
Age (years)	23.5 ± 0.6
Weight (kg)	75.7 ± 1.9
Height (cm)	171.2 ± 1.7
Body mass index (BMI; kg/m^2^)	25.7 ± 0.7
Fasting plasma glucose (mg/dL)	88.7 ± 1.7
Total cholesterol (mg/dL)	190.1 ± 8.7
Serum triglyceride (mg/dL)	97.9 ± 12.6
Creatinine (mg/dL)	1.06 ± 0.04
Blood urea nitrogen (mg/dL)	14.7 ± 0.8
Aspartate transaminase (U/L)	23.9 ± 2.0
Alanine transaminase (U/L)	33.7 ± 6.3

All values are means ± SEM, *n* = 16.

## Data Availability

The data presented in this study are available on reasonable request from the corresponding author C.C. (charoonsri.c@chula.ac.th).
